# Association between objectively measured physical activity and maternal stool microbiota during pregnancy: results from a preliminary investigation

**DOI:** 10.3389/fcimb.2026.1747305

**Published:** 2026-04-23

**Authors:** Sara Santarossa, Alexandra R. Sitarik, Faith L. Houck, Clinton A. Brawner, Christine Cole Johnson, Sarah S. Comstock, Susan V. Lynch, Paula M. van Wyk, Faith Vundla, Claire Schachtschneider, Jennifer K. Straughen, Andrea E. Cassidy-Bushrow

**Affiliations:** 1 Department of Public Health Sciences, Henry Ford Health, Detroit, MI, United States; 2 Department of Obstetrics, Gynecology and Reproductive Biology, College of Human Medicine, Michigan State University, East Lansing, MI, United States; 3 Henry Ford Health + Michigan State University Health Sciences, Detroit, MI, United States; 4 Department of Epidemiology and Biostatistics, College of Human Medicine, Michigan State University, East Lansing, MI, United States; 5 Department of Kinesiology, Michigan State University, East Lansing, MI, United States; 6 Division of Cardiovascular Medicine, Henry Ford Hospital & Medical Group, Henry Ford Health, Detroit, MI, United States; 7 Department of Food Science and Human Nutrition, Michigan State University, East Lansing, MI, United States; 8 Benioff Center for Microbiome Medicine, Department of Medicine, University of California San Francisco, San Francisco, CA, United States; 9 Department of Kinesiology, University of Windsor, Windsor, ON, Canada; 10 Department of Pediatrics and Human Development, College of Human Medicine, Michigan State University, East Lansing, MI, United States

**Keywords:** bacteria, exercise, microbiota, physical activity, pregnancy

## Abstract

**Background:**

This study investigated the relationship between objectively measured physical activity (PA) and gut microbiota composition during pregnancy.

**Methods:**

In an ongoing birth cohort study, the Fitbit Charge wearable activity monitor was used to objectively measure PA during pregnancy. To profile the composition of gut bacterial microbiota, 16S rRNA sequencing was performed on a third trimester stool sample. Differences in alpha diversity metrics (richness, Pielou’s evenness, and Faith’s phylogenetic diversity) by PA were determined using linear regression, whereas beta diversity (unweighted and weighted UniFrac, Canberra, and Bray-Curtis) relationships were assessed using permutational multivariate analysis of variance (PERMANOVA). Differential abundance testing at both the amplicon sequence variants and genus level was conducted using Analysis of Compositions of Microbiomes with Bias Correction 2; tests were corrected for false discovery rate (FDR) and considered significant if p_FDR_ < 0.05.

**Results:**

The analytic sample included 68 pregnant women with both PA and 16S rRNA sequencing data (median age [quartile 1; quartile 3] = 30.7 [26.6; 33.3] years; 56% Black). Women typically took around 5000 steps per day (second trimester median = 5486; third trimester median = 5074) and the majority of activity minutes were classified as sedentary (second trimester median = 77%; third trimester median = 78%). None of the PA variables during the second or third trimester were associated with gut microbiota richness, evenness, or diversity. After covariate adjustment, the proportion sedentary during the second (Weighted UniFrac p = 0.035, R^2^ = 0.038) and third (Bray-Curtis p = 0.034, R^2^ = 0.025) trimesters were significantly associated with stool microbiota composition. Additionally, the proportion “fairly” or “very active” during the third trimester was significantly associated with stool microbiota composition (PERMANOVA; Bray-Curtis p = 0.047, R^2^ = 0.025). These compositional differences were partly characterized by significantly lower abundances of *Prevotella* and *Pasteurellaceae* ASVs and higher abundances of *Acidaminococcus* and *Finegoldia* in pregnant women with higher second and third trimester sedentary time (p_FDR_ < 0.05). Two specific 16S rRNA sequence variants (ASV_84 [*Acidaminococcus intestine*] and ASV_134 [*Oscillospirales* sp.]) were also positively associated with sedentary behavior. No significant microbial changes were observed with the proportion of time spent “fairly” or “very active” during the third trimester.

**Conclusions:**

These findings highlight sedentary behavior as a potential modifiable factor related with maternal gut microbiota composition during pregnancy. This study forms the basis for future studies on the impact of sedentary time on gut microbiota during pregnancy.

## Introduction

Being physically active is encouraged during pregnancy, with current guidelines recommending at least 150 minutes of aerobic physical activity (PA) at moderate intensity each week, unless contraindicated (Mottola et al., 2018; Piercy et al., 2018). PA encompasses both deliberate exercise and routine daily movements (American College of Sports Medicine, 2014). Engaging in PA during pregnancy has been linked to a range of improved outcomes for both mother and child, including a lower likelihood of excessive gestational weight gain or gestational diabetes mellitus (da Silva et al., 2017; Piercy et al., 2018; Wang et al., 2019; Morales-Suárez-Varela et al., 2020). Additionally, PA is known to enhance mental health (Prather et al., 2012), support better sleep (Guerin et al., 2018), and lessen the severity of postpartum depression symptoms (Piercy et al., 2018).

Beyond pregnancy outcomes, PA has also been associated with several *in utero* benefits, such as optimal fetal heart rate, better placental function, and increased levels of amniotic fluid (Prather et al., 2012; Davenport et al., 2018a). These prenatal benefits may extend beyond birth, contributing to reduced risk of macrosomia (Prather et al., 2012; Piercy et al., 2018), improved early neurodevelopment (Prather et al., 2012; Morales-Suárez-Varela et al., 2020), and a lower adiposity during childhood (Prather et al., 2012; Morales-Suárez-Varela et al., 2020). Despite these well-documented associations, only 14% of pregnant persons in the United States meet the recommended PA guidelines (Rodrigues-Denize et al., 2024). Furthermore, the specific biological mechanisms through which PA positively impacts pregnancy outcomes remain largely unclear (Dipietro et al., 2019).

Several physiological and psychological pathways have been proposed to explain how PA during pregnancy may lead to better pregnancy and birth outcomes. These include improved metabolic function (Davenport et al., 2018b; Adamo et al., 2024), reduced inflammation (Dhar et al., 2024; Wisseman et al., 2025), enhanced cardiovascular health (Davenport et al., 2018b; Barakat et al., 2023; McLean et al., 2025), hormonal balance (Adamo et al., 2024), and better psychological regulation (Cai et al., 2022; Adamo et al., 2024; Rim et al., 2025). In addition to these more established mechanisms, recent interest has emerged around the gut microbiome as a potentially important, but less-explored, pathway through which PA could influence maternal and fetal health (Cerda et al., 2016; Campaniello et al., 2022; Cataldi et al., 2022).

During pregnancy, the gut microbiome undergoes substantial remodeling (Koren et al., 2012). While studies of prenatal gut microbiome in early pregnancy are limited, they suggest that microbial composition is relatively unchanged at the beginning of pregnancy, but substantial compositional shifts are seen by the third trimester (Sinha et al., 2023). This is thought to be a result of hormonal changes or low-grade inflammation (Sinha et al., 2023). The remodeling of the gut microbiome during pregnancy plays a role in shaping early life gut microbiota composition in offspring (Wang et al., 2021) and is therefore an emerging modifiable factor of interest for infant health. Though gut microbial communities are highly personalized, it has been previously shown that up to 20% of its interindividual variation can be explained by various personal and environmental factors (Falony et al., 2016). Prenatal PA may be an underexplored exposure that shapes prenatal gut microbiome composition.

Despite studies in non-pregnant populations showing PA alters the gut microbiome (Langsetmo et al., 2019; Mitchell et al., 2019; Ramos et al., 2022), there are limited data during pregnancy. Emerging evidence from both human and rodent models suggests that prenatal PA may influence gut microbial composition; however, findings remain inconsistent. In rodent studies, prenatal PA has been shown to be associated with increased abundance of potentially beneficial taxa (e.g., *Bifidobacterium pseudolongum* (Duan et al., 2022), *Odoribacter* (Zhou et al., 2020), and *Clostridium* (Zhou et al., 2020; Hinrichs et al., 2023)) and reduced abundance of taxa implicated in inflammation or metabolic dysfunction (e.g., *Desulfovibrio* (Duan et al., 2022) and *Escherichia/Shigella* (Duan et al., 2022)). Recent research in humans found that those who met the prenatal PA guidelines presented with lower abundances of Bacteroidales (Santarossa et al., 2023), which has been previously correlated with a lower risk of negative maternal health outcomes (e.g., preeclampsia) (Wang et al., 2020). Neither of these studies used an objective measure of PA; subjective measures such as self-reports via diaries or recall surveys may overestimate the PA performed, both in intensity and duration (Dishman et al., 2001). Thus, in this study, we aimed to explore the association of objective PA, collected via a Fitbit (Google, Mountainview, CA, USA) wearable activity monitor, during pregnancy on the prenatal gut microbiota composition in a subgroup of participants from the Research Enterprise to Advance Children’s Health (REACH) birth cohort, REACH-Fitbit.

## Materials and methods

### Study population

Details of the REACH-Fibit sub-cohort and REACH cohort have been published (Lyons et al., 2023; Santarossa et al., 2024; Redding et al., 2025; Santarossa et al., 2025). In brief, REACH aims to recruit racially, ethnically, culturally, and socioeconomically diverse maternal-child pairs from the metropolitan Detroit area to investigate a range of exposures and health outcomes. To be eligible for REACH, participants must be 18 years or older, receiving care at 1 of 28 prenatal care sites, and planning to deliver at 1 of 4 Henry Ford Health hospitals. There are no exclusion criteria for comorbid conditions or prior medical history; thus cohort participants are not selected for high-risk conditions and better represent the general obstetric population. Only one baby per pregnant person and pregnancy can be enrolled into the study (e.g., only 1 baby out of a set of twins can be enrolled). All participants in REACH recruited before 20 weeks of gestation and that have a Bluetooth-enabled device (e.g., smart phone, tablet) to synchronize data with the Fitbit Application are eligible for REACH-Fitbit. Recruitment for REACH-Fitbit began in February 2022 and is ongoing. The following analysis includes sub-cohort participants with available data through May 1, 2024. This study was approved by the Henry Ford Health Institutional Review Board (#13198-01; August 28, 2019).

### Fitbit study protocol

REACH-Fitbit participants were provided a Fitbit Charge (models 4, 5, or 6) during a mid-pregnancy (before 24 weeks gestation) research visit. At this research visit, study team members supported participants in creating a Fitbit account and authorizing the Henry Ford Health Fitbit Tool (HFH-FiT) to harvest data from the Fitbit API (Santarossa et al., 2025). Participants were instructed to wear the device on their non-dominant wrist, sync daily, and continue their daily activities as usual. Fitbit data collection ceases following parturition. Participants keep their Fitbit following study completion. Compliance to the REACH-Fitbit study protocol (Santarossa et al., 2025) is measured using intraday HR to capture daily wear time, with HR measured every 15 minutes. Compliance (valid day) is defined as HR data for ≥ 10 hours/day (Speier et al., 2018; Marques et al., 2021). An overall compliance percentage is calculated as the ratio of compliant days to total days enrolled in the study.

### Prenatal PA assessment

The Fitbit Charge is a commercially available mid-range, wrist-worn, wearable activity monitor, capable of numerous PA monitoring techniques. For this study, a variety of exposure variables were calculated using Fitbit data. In both the second and third trimesters, average daily steps were calculated using all days of valid data (worn ≥ 10 hours/day) within each trimester, for each participant. Additionally, the Fitbit technology uses metabolic equivalents (METs), a unit used to quantify the energy expenditure of PA, calculates active minutes, and reports them as “sedentary minutes”, ‘‘lightly active minutes”, ‘‘fairly active minutes”, and ‘‘very active minutes”. The proportion of all activity minutes that were categorized as sedentary (< 1.5 METs), lightly active (1.5–3 METs), fairly active (i.e., moderate activity) (3–6 METs), or very active (i.e., vigorous activity) (> 6 METs) within each trimester (all days of valid data) was calculated. In other words, each participant has a continuous percentage value for each MET category in each trimester. For analysis purposes, fairly or very active were combined into 1 category (> 3 METs) due to sparse distributions.

### Stool specimen collection

REACH participants were asked to provide a stool sample in their third trimester (between 28 weeks gestation to birth). Participants were provided with a stool swab sample collection kit (Protocult Collection Device paper sample collector, Ability Building Center, Rochester, MN, USA; a Simport Cryovial, Beloeil, QC, Canada collection tube containing DNA/RNA Shield Reagent, Zymo Research Corporation, CA, USA; Copan Diagnostics Nylon Flocked Dry Swabs in Peel Pouches, Murrieta, CA, USA) to collect a stool swab for analysis. The collection kits were distributed, via postal mail, with detailed sample collection instructions. All stool samples retrieved from the participants and delivered to the research facility were considered viable. The stool samples were then aliquoted and stored at -80 °C. Stool swab aliquots were shipped by overnight shipping on dry ice to the University of California, San Francisco, where 16S rRNA sequencing was performed, as described below.

### DNA extraction

Stool samples underwent DNA extraction conducted by the Microbial Genomics Facility at the University of California, San Francisco. The DNA extraction process utilized a modified cetyltrimethylammonium bromide (CTAB) buffer-based protocol, as detailed in a published article (Fujimura et al., 2016). Briefly, the stool samples in Zymo preservative (DNA/RNA Shield) were thawed on ice. Each sample was vortexed thoroughly, then 300 µLs of each sample was added into Lysing Matrix E tube (MP Biomedicals, Irvine, CA, USA) containing 500 µLs of 5% CTAB extraction buffer. Samples were then incubated at 65 °C for 15 minutes. After incubation, 500 µLs of phenol:chloroform:isoamyl alcohol (25:24:1) was added, followed by bead-beating at 5.5 m/s for 30 seconds. Samples are then centrifuged at 16,000xg for 5 minutes at 4 °C. The resulting aqueous phase (~400 µL) was transferred to a new 2-mL Eppendorf tube containing 800 µL of chloroform. An additional 400 uLs of 5% CTAB buffer was added to the remaining organic material in the Lysing Matrix E tubes followed by another 30 second bead-beating and 5-minute centrifugation step as described above. The resulting aqueous phase (~500 µL) was transferred to the corresponding 2-mL Eppendorf tube containing 800 µL of chloroform and first aqueous phase. The aqueous phase and chloroform were thoroughly mixed then centrifuged at 16,000xg for 5 minutes at 4 °C. The aqueous layer was carefully pipetted into a new Eppendorf tube and combined with 1:1 by volume of 30% polyethylene glycol/NaCl solution and stored at 4 °C overnight to precipitate DNA. After overnight incubation, samples were centrifuged (16,000xg for 10 minutes), washed twice with freshly prepared, ice-cold, molecular-grade 70% ethanol. A negative extraction control was included in each extraction batch to account for potential contaminants in reagents. The extracted DNA from each sample was quantified using the Qubit 2.0 Fluorometer with the dsDNA BR Assay Kit (Life Technologies, Grand Island, NY) and normalized to 5 ng/µL using QIAgility Liquid Handler (QIAGEN, Hilden, Germany).

### Amplicon library preparation and sequencing

The hypervariable region 4 (V4) of the 16S rRNA gene was amplified via polymerase chain reaction using 515F/806R primers. The reverse primers (806R) contained a unique barcode sequence to enable demultiplexing of pooled samples and an adapter sequence that enabled the amplicons to bind to the flow cell. Each sample was amplified in a 25-μL reaction using 0.025 U Takara Hot Start ExTaq (Takara Mirus Bio Inc., Madison, WI, USA), 1X Takara buffer, 0.4 pmol μL−1 of F515 and R806 primers, 0.56 mg mL^−1^ of bovine serum albumin (Roche Applied Science, Indianapolis, IN, USA), 200 μM of deoxyribonucleotide triphosphates, and 10 ng of template DNA. The polymerase chain reaction conditions consisted of an initial denaturation at 98 °C for 2 minutes, followed by 30 cycles at 98 °C for 20 seconds, 50 °C for 30 seconds, and 72 °C for 45 seconds, concluding with a final extension at 72 °C for 10 minutes. Post-amplification, the amplicons were quantified using the Qubit 2.0 Fluorometer with a dsDNA HS Assay Kit (Life Technologies) and confirmed via 2% tris-borate-EDTA agarose e-gel (Life Technologies). The amplicons were pooled at equimolar concentration using the liquid handler (Opentrons OT-2; Opentrons Labworks Inc., Long Island City, NY, USA), and purified using AMPure SPRI beads (Beckman Coulter, Brea, CA, USA). The final amplicons library quality and quantity were assessed with the Bioanalyzer DNA 1000 Kit (Agilent, Santa Clara, CA, USA) and the Qubit 2.0 Fluorometer with a dsDNA HS Assay Kit, respectively. The final pooled amplicons library was diluted to 2 nM, PhiX spike-in control was added at a 10% final concentration, and the samples were sequenced on the Illumina MiSeq Platform (San Diego, CA, USA) producing paired-end 280 x 280 bp reads.

### Sequencing data processing

The Illumina MiSeq generated paired-end 280bp sequencing data were demultiplexed using unique barcodes and converted into fastq format via the bcl2fastq software. Forward and reverse reads were processed separately and quality-filtered using the DADA2 software package (v1.16.0) in R 4.1.2 (Callahan et al., 2016). Reads with more than 2 expected errors were discarded, and sequences were truncated to 230 bp and 200 bp for forward and reverse reads, respectively, using the filterAndTrim function in DADA2 to retain high-quality reads for denoising. Error rates of the filtered dereplicated reads were estimated using 100,000 sequences. Paired reads with a minimum overlap of 25 bp were merged to obtain full denoised sequences. Amplicon sequence variants (ASVs) were inferred exactly, resolving even single-nucleotide differences (i.e., 100% sequence identity). Chimeric ASVs were identified and removed. Taxonomic assignment was performed using the assignTaxonomy function in DADA2, with an 80% bootstrap cutoff, applying the naïve Bayesian classifier method and the SILVA v138 16S rRNA reference database. A phylogenetic tree was constructed using the phangorn and DECIPHER packages (Quast et al., 2013). The ASV table was then filtered to retain only variants classified under the kingdom Bacteria. Lastly, a rarefied ASV table was generated by representative rarefying to the minimum depth of 20,340 sequences per sample, which was used in conjunction with the unrarefied ASV table for downstream analyses.

### Potential confounders

Demographic and obstetric information were obtained from participants’ electronic medical records. Questionnaires were administered throughout pregnancy using REDCap (Vanderbilt University, Nashville, TN, USA) (Harris et al., 2009; Harris et al., 2019). Maternal age at start of pregnancy and ethnicity were obtained from the electronic medical record; if missing, questionnaire data were used. Race, marital status, parity, and pre-pregnancy body mass index were also obtained from the electronic medical record. Education and household income were obtained from prenatal questionnaires.

Prenatal dietary quality, measured using the Healthy Eating Index (0–100 scale), was captured using the Diet ID survey instrument (Katz et al., 2020; Diet ID, 2022a; Diet ID, 2022b; Santarossa et al., 2024).

### Statistical analysis

Statistical significance was pre-specified at 0.05. All analyses were conducted in R version 4.3.1. All PA variables were treated as continuous throughout. Descriptive statistics were first generated to understand data distributions. The association between potential confounders and PA variables were evaluated using the Kruskal-Wallis test for categorical covariates and Pearson correlations for continuous covariates. To evaluate the association between potential confounders and beta diversity (calculated using unweighted and weighted UniFrac, Canberra, and Bray-Curtis distances), permutational multivariate analysis of variance (PERMANOVA) tests with 1000 permutations were utilized (Anderson, 2001). Given the limitations of the dataset (small sample size and some covariates with high missingness), potential confounders were selected for model adjustment if they were associated with both PA and microbiota composition (i.e., beta diversity). However, the fully adjusted model was also fit as a sensitivity analysis. To evaluate the association between PA and alpha diversity (richness, Pielou’s evenness, and Faith’s phylogenetic diversity), linear regression models were used, with each alpha diversity metric as the outcome and each individual PA variable as the exposure (per 1-SD increase). To evaluate the association between PA and beta diversity, PERMANOVA testing was performed as we have previously described (Özçam et al., 2025). Beta dispersion tests were not performed due to the ambiguity in how this assumption should be evaluated with continuous variables. The first four principal coordinates (PCOs) of each beta diversity metric were derived and plotted to visualize associations. Differential abundance testing at both the ASV and genus level was conducted using Analysis of Compositions of Microbiomes with Bias Correction 2 (ANCOM-BC2) (Lin and Peddada, 2020); tests were corrected for false discovery rate (FDR) and considered significant if p_FDR_ < 0.05. For both alpha and beta diversity, the rarefied ASV table was used, whereas for differential abundance testing, the unrarefied ASV table was used, as read depth is accounted for in ANCOM-BC2. PA variables were subjected to differential abundance testing if at least 1 significant adjusted PERMANOVA test was found (p < 0.05).

## Results

Recruitment for REACH-Fitbit began in February 2022 and is ongoing. At the time of data curation for the current manuscript (May 1, 2024), a total of 130 women had complete Fitbit data and at least 1 day of valid data (worn 10+ hours per day). Of these 130 women, a total of 68 had a third trimester stool sample sequenced for microbiota. The majority of women had Fitbit data during both the second and third trimesters (N = 59), but 5 women had second trimester Fitbit data only, and 4 had third trimester Fitbit data only. Descriptive statistics of the 68 women included in the analysis subset are shown in **Table 1**. The median number of days of valid data during the second trimester was 45 ([Q1; Q3] = [15; 64]), while the median number of days of valid data during the third trimester was 50 ([Q1; Q3] = [24.5; 70]). The majority of women were Black (56%), not Hispanic/Latino (88%), unmarried (60%), had household incomes < $50,000 (56%), and had parity > 0 (68%). Women typically took around 5000 steps per day (second trimester median = 5486; third trimester median = 5074) and the majority of activity minutes were classified as sedentary (second trimester median = 77%; third trimester median = 78%; **Table 1**).

**Table 1:** Description of REACH women included in analyses (N = 68).

Variable	N	Median [Q1; Q3] or N (%)
Maternal characteristics		
Age at the start of pregnancy (years)	68	30.7 [26.6; 33.3]
Race	68	
Black		38 (56%)
White		20 (29%)
Other/Multiracial		10 (15%)
Ethnicity	68	
Hispanic or Latino		8 (12%)
Not Hispanic/Latino		60 (88%)
Married	68	
No		41 (60%)
Yes		27 (40%)
Education	40	
Some college or less		20 (50%)
Bachelor’s degree or more		20 (50%)
Household income	45	
<$50,000		25 (56%)
≥$50,000		20 (44%)
Parity	68	
0		22 (32%)
1+		46 (68%)
Pre-pregnancy BMI (kg/m^2^)	53	27.0 [24.1; 32.8]
HEI	43	64.0 [44.5; 80.0]
PA during second trimester		
Number of days of valid data	64	45 [15; 64]
Average steps	64	5486 [4389; 7677]
Percent sedentary	64	77% [72%; 82%]
Percent lightly active	64	22% [17%; 25%]
Percent fairly or very active	64	1% [0%; 2%]
PA during third trimester		
Number of days of valid data	63	50 [24.5; 70]
Average steps	63	5074 [3726; 6457]
Percent sedentary	63	78% [74%; 83%]
Percent lightly active	63	21% [17%; 24%]
Percent fairly or very active	63	0% [0%; 1%]

BMI, body mass index; HEI, Health Eating Index; PA, physical activity; Q1, quartile; Q3, quartile; REACH, Research Enterprise to Advance Children’s Health.

### Factors associated with PA and microbiota composition

To determine the set of adjustment covariates among all potential confounding factors (shown under “maternal characteristics” in **Table 1**), we examined which covariates were associated with PA and stool microbiota composition. Age at the start of pregnancy, race, marital status, education, and pre-pregnancy body mass index were each significantly associated with at least 1 PA variable in the second or third trimester (**Supplementary Figure 1**; p < 0.05). Race, marital status, and parity were significantly associated with stool microbiota beta diversity (**Supplementary Figure 2**; p < 0.05). Therefore, both race and marital status were included in adjusted models for PA/microbiota associations.

### Relationships between PA and microbiota diversity

When the association between PA and stool alpha diversity was examined (**Table 2**), no significant associations were found in either unadjusted (all p ≥ 0.10) or adjusted (all p ≥ 0.18) models.

**Table 2:** Association between PA during the second or third trimester and stool alpha diversity during the third trimester.

Alpha diversity metric	PA trimester^a^	PA variable	Unadjusted		Adjusted^c^	
			β (95% CI)^b^	p-value	β (95% CI)^b^	p-value
Richness	Second	Average steps	1.80 (-10.01 to 13.61)	0.76	2.07 (-10.29 to 14.43)	0.74
		Proportion sedentary	-1.62 (-13.43 to 10.19)	0.79	-1.74 (-14.23 to 10.76)	0.78
		Proportion lightly active	1.90 (-9.90 to 13.71)	0.75	2.06 (-10.31 to 14.43)	0.74
		Proportion fairly or very active	-0.63 (-12.44 to 11.19)	0.92	-0.86 (-13.26 to 11.54)	0.89
	Third	Average steps	0.14 (-11.70 to 11.97)	0.98	0.13 (-11.94 to 12.20)	0.98
		Proportion sedentary	-3.61 (-15.41 to 8.18)	0.54	-2.66 (-15.30 to 9.99)	0.68
		Proportion lightly active	2.16 (-9.66 to 13.98)	0.72	1.33 (-11.12 to 13.79)	0.83
		Proportion fairly or very active	9.63 (-1.94 to 21.20)	0.10	8.72 (-4.05 to 21.48)	0.18
Evenness	Second	Average steps	0.001 (-0.014 to 0.015)	0.94	-0.000 (-0.015 to 0.014)	0.98
		Proportion sedentary	-0.002 (-0.017 to 0.012)	0.73	-0.001 (-0.015 to 0.014)	0.90
		Proportion lightly active	0.003 (-0.011 to 0.017)	0.67	0.002 (-0.012 to 0.016)	0.77
		Proportion fairly or very active	-0.002 (-0.016 to 0.012)	0.81	-0.004 (-0.018 to 0.011)	0.60
	Third	Average steps	0.002 (-0.012 to 0.017)	0.77	0.002 (-0.013 to 0.016)	0.83
		Proportion sedentary	-0.004 (-0.019, 0.010)	0.55	-0.002 (-0.017 to 0.014)	0.83
		Proportion lightly active	0.004 (-0.010 to 0.019)	0.55	0.002 (-0.013 to 0.017)	0.80
		Proportion fairly or very active	0.002 (-0.013 to 0.016)	0.80	-0.002 (-0.018 to 0.014)	0.82
Phylogenetic diversity	Second	Average steps	0.06 (-1.38 to 1.50)	0.94	0.09 (-1.42 to 1.60)	0.90
		Proportion sedentary	-0.07 (-1.51 to 1.37)	0.93	-0.10 (-1.63 to 1.43)	0.90
		Proportion lightly active	0.20 (-1.24 to 1.64)	0.78	0.24 (-1.27 to 1.75)	0.75
		Proportion fairly or very active	-0.44 (-1.88 to 1.00)	0.54	-0.47 (-1.98 to 1.04)	0.54
	Third	Average steps	-0.04 (-1.45 to 1.37)	0.95	-0.04 (-1.49 to 1.40)	0.95
		Proportion sedentary	-0.35 (-1.75 to 1.06)	0.62	-0.25 (-1.76 to 1.27)	0.75
		Proportion lightly active	0.23 (-1.18 to 1.64)	0.75	0.15 (-1.34 to 1.64)	0.84
		Proportion fairly or very active	0.81 (-0.58 to 2.21)	0.25	0.66 (-0.88 to 2.20)	0.39

PA, physical activity.

^a^ N=64 for all second trimester models; N = 63 for all third trimester models.

^b^ Interpreted as the change in alpha diversity metric for each 1-standard deviation increase in the specified physical activity variable.

^c^ Adjusted for race and marital status.

### Relationships between PA and microbiota composition

Next, we evaluated the association between PA and stool microbiota composition using phylogenetic beta diversity metrics (**Table 3**). After adjusting for race and marital status, an association was found for proportion sedentary during the second trimester (Weighted UniFrac p = 0.035, R^2^ = 0.038; **Supplementary Figure 3**). When non-phylogenetic metrics were examined, proportion sedentary (Bray-Curtis p = 0.034, R^2^ = 0.025) and proportion fairly or very active (**Supplementary Figures 3**, **4**; Bray-Curtis p = 0.047, R^2^ = 0.025) during the third trimester were both significantly associated with stool microbiota composition (**Supplementary Table 1**; **Supplementary Figure 4**). These data indicate that the gut microbiotas of participants with less sedentary time were compositionally distinct from those with greater sedentary time (**Supplementary Figures 3**, **4**). In sensitivity analyses fully adjusting for all nine potential confounders, roughly a third of the sample size was included due to missingness in covariates (**Supplementary Table 2**). Though no tests reached statistical significance with this reduced sample size, a relatively large proportion of variance was nonetheless explained by some PA variables, such as proportion fairly or very active during the third trimester (Bray-Curtis p=0.064, R^2^ = 0.062).

**Table 3:** Association between PA during the second and third trimester and stool beta diversity during the third trimester.

Beta diversity metric	PA trimester^a^	PA variable	Unadjusted		Adjusted^b^	
			p-value	R^2^	p-value	R^2^
Unweighted UniFrac	Second	Average steps	0.54	0.015	0.60	0.014
		Proportion sedentary	0.16	0.019	0.24	0.018
		Proportion lightly active	0.17	0.019	0.21	0.018
		Proportion fairly or very active	0.66	0.014	0.83	0.012
	Third	Average steps	0.32	0.017	0.44	0.016
		Proportion sedentary	**0.030**	0.027	0.10	0.022
		Proportion lightly active	0.058	0.024	0.14	0.02
		Proportion fairly or very active	0.076	0.023	0.24	0.018
Weighted UniFrac	Second	Average steps	0.81	0.008	0.93	0.006
		Proportion sedentary	**0.013**	0.053	**0.035**	0.038
		Proportion lightly active	**0.021**	0.043	0.060	0.032
		Proportion fairly or very active	0.19	0.023	0.42	0.015
	Third	Average steps	0.42	0.015	0.60	0.011
		Proportion sedentary	**0.033**	0.047	0.059	0.035
		Proportion lightly active	**0.044**	0.041	0.095	0.03
		Proportion fairly or very active	0.091	0.03	0.14	0.026

PA, physical activity.

^a^ N=64 for all second trimester models; N = 63 for all third trimester models.

^b^ Adjusted for race and marital status.

Bold values denote statistical significance at the p<0.05 level.

### Relationships between PA and gut bacterial relative abundance

When differential abundance testing was conducted with counts collapsed at the genus level (or higher of genus level classification was unavailable), a total of 4 taxa reached statistical significance for increasing the proportion sedentary during the second and third trimesters after covariate adjustment (**Table 4**, p_FDR_ < 0.05): *Prevotella*, *Acidaminococcus*, *Finegoldia*, and *Pasteurellaceae*. Specifically, increasing the proportion sedentary during the second and third trimesters were associated with decreasing *Prevotella* abundance; increasing the proportion sedentary during the second trimester was also associated with decreasing abundance of *Pasteurellaceae*. Conversely, increasing the proportion sedentary during the second and third trimesters were associated with increasing *Acidaminococcus* abundance; increasing the proportion sedentary during the second trimester was associated with increasing *Finegoldia* abundance. Differential abundance testing was also conducted for the proportion fairly or very active during the third trimester, but no genera reached statistical significance.

**Table 4:** Third trimester stool genera significantly associated with proportion sedentary (p_FDR_ < 0.05).

PA variable	Genus^a^	LFC^b^	SE	p_FDR_
Proportion sedentary during second trimester	*Prevotella*	-15.40	3.62	0.011
	*Acidaminococcus*	14.22	2.77	0.012
	*Finegoldia*	17.52	2.73	0.018
	*Pasteurellaceae*	-11.05	2.63	0.020
Proportion sedentary during third trimester	*Prevotella*	-18.57	3.45	0.001
	*Acidaminococcus*	13.27	2.72	0.014

FDR, false discovery rate; LFC, Log Fold Change; PA, physical activity; SE, Standard Error.

^a^ tests are collapsed to the genus level, or a higher taxonomic level if genus level classification is not available.

^b^ Adjusted for race and marital status.

When differential abundance testing was conducted at the ASV level, 2 ASVs reached statistical significance (**Table 5**). Specifically, increasing the proportion sedentary during the second and third trimesters was associated with increasing abundance ASV_84 (*Acidaminococcus intestini*), while increasing the proportion sedentary during the third trimester was associated with increasing abundance ASV_134 (*Oscillospirales* sp.). Again, no ASVs reached statistical significance for the proportion fairly or very active during the third trimester.

**Table 5:** Third trimester stool Amplicon sequence variants (ASVs) significantly associated with proportion sedentary (pFDR < 0.05).

PA variable	Taxon	Phylum	Class	Order	Family	Genus	Species	LFC	SE	p_FDR_
Proportion sedentary during second trimester	ASV_84	*Firmicutes*	*Negativicutes*	*Acidaminococcales*	*Acidaminococcaceae*	*Acidaminococcus*	*intestini*	15.73	2.82	0.033
Proportion sedentary during third trimester	ASV_134	*Firmicutes*	*Clostridia*	*Oscillospirales*	NA	NA	NA	16.59	2.72	0.033
	ASV_84	*Firmicutes*	*Negativicutes*	*Acidaminococcales*	*Acidaminococcaceae*	*Acidaminococcus*	*intestini*	14.30	2.79	0.033

FDR, false discovery rate; LFC, Log Fold Change; NA, Not Applicable; PA, physical activity; SE, Standard Error.

## Discussion

In this preliminary analysis of the effect of objective PA on the gut microbiota of pregnant women, we found that measures of PA during the second and third trimester were associated with third trimester gut microbiota composition. Specifically, greater sedentary behavior was associated with distinct microbial profiles, decreased abundance of *Prevotella* and *Pasteurellaceae*, and increased abundance of *Acidaminococcus* and *Finegoldia*. These results suggest that sedentary time may be a driver of gut microbial variation during pregnancy. Moreover, sedentary behavior was positively associated with 2 specific amplicon sequence variants (ASVs): *Acidaminococcus intestini* (ASV_84) during both trimesters and *Oscillospirales* sp. (ASV_134) during the third trimester. These findings support the notion that increased sedentary time is associated with compositional changes in gut microbial communities that may have metabolic implications (Xu et al., 2022). While few studies have examined prenatal PA in relation to the maternal microbiome, our findings contribute to a growing body of literature. Prior human studies have shown that pregnant individuals who met or exceeded PA guidelines had a lower relative abundance of *Bacteroidales*, *Bifidobacteriaceae*, *Lactobacillaceae*, and *Streptococcaceae* (Santarossa et al., 2023). Additionally, positive associations have been observed between PA and beneficial taxa such as *Prevotellaceae UCG-001*, particularly among Malaysian pregnant individuals with gestational diabetes (Kunasegaran et al., 2024). While cross-study comparisons are limited by methodological differences, these findings collectively support the role of PA, and sedentary behavior in particular, as a modifiable factor influencing the maternal gut microbiome.

The observed inverse relationship between sedentary time and *Prevotella* is particularly compelling, given prior associations between *Prevotella* and favorable metabolic profiles. *Prevotella*, is a genus commonly linked to high-fiber diets and traditional (non-Western) dietary intake (Prasoodanan et al., 2021; Maher et al., 2023). Its presence is often interpreted as beneficial, associated with improved glucose metabolism, increased short-chain fatty acid production, and reduced inflammation. The inverse relationship between *Prevotella* and sedentary behavior aligns with prior findings in both pregnant and non-pregnant populations where increased PA or exercise interventions were associated with higher *Prevotella* abundance (Romberg et al., 2004; Petersen et al., 2017; Dziewiecka et al., 2022).

The taxa enriched in association with sedentary behavior, particularly *Acidaminococcus intestini* and *Finegoldia*, have been implicated in metabolic disorders and inflammatory responses. These findings may have implications for pregnancy outcomes. *Acidaminococcus intestini* is known to be involved in protein fermentation and may contribute to the production of branched-chain fatty acids, which have been implicated in insulin resistance (Shah et al., 2024). Its enrichment with greater sedentary time could suggest a shift away from carbohydrate- and fiber-utilizing microbes toward taxa associated with less favorable metabolic profiles. While speculative, these pathways may represent one mechanism by which sedentary behavior contributes to pregnancy complications such as gestational diabetes or preeclampsia. Similarly, *Finegoldia*, a pathobiont often associated with inflammatory states (Neumann et al., 2020), was positively associated with sedentary behavior in the second trimester, while *Pasteurellaceae*, which includes commensal gastrointestinal bacteria (Crosby and Woolums, 2022), showed negative associations. The presence of *Oscillospirales* sp., a member of the Firmicutes phylum, is more ambiguous. Some species within this group are short-chain fatty acid producers and generally considered beneficial; however, their abundance can also be shaped by broader metabolic and inflammatory states (Fusco et al., 2023). Its positive association with sedentary time raises questions about the nuanced role of lifestyle factors in modulating microbial function, not just composition.

Notably, we did not observe significant associations between the proportion of time spent in fairly or very active minutes and microbiota at the taxonomic level, despite modest associations with beta diversity. This may reflect several factors. First, our cohort engaged in relatively low levels of moderate-to-vigorous activity, which could limit the statistical power to detect meaningful associations. Second, the corrections for multiple comparisons in differential abundance testing require strong effect sizes, which may not have been present. Third, it is possible that sedentary behavior exerts a stronger or more consistent influence on gut microbial composition than short periods of higher-intensity activity. This aligns with prior literature suggesting that breaking up sedentary time may have independent metabolic benefits (Healy et al., 2008; Thyfault and Bergouignan, 2020), potentially mediated through distinct microbial pathways. Future work should examine whether interventions to reduce sedentary time, not just increase active time, can beneficially shift the maternal microbiome.

### Strengths and limitations

Our study also has several strengths, the most notable being that objective PA during pregnancy was captured through wearable devices rather than by assessment using self-report questionnaires. This is the first study to our knowledge to assess the association between prenatal PA and microbiome using objective PA data. It is well known that measurement error in subjective PA assessments is common, with PA levels typically being overreported (Dishman et al., 2001). This has also been shown to be true among pregnant populations (Sattler et al., 2018). Additionally, a diverse cohort of pregnant participants was used for analysis, as over half of those included were Black, increasing the generalizability of findings and addressing an important gap in the microbiome literature.

However, several limitations must be noted. First, PA levels during pregnancy in this birth cohort study were observational in nature and were not randomly assigned; participants were encouraged to wear their device, rather than encouraged to alter their PA levels. Therefore, unmeasured or residual confounding is possible. However, assuming confounding has been adequately controlled for, the real-world data collection utilized here allows us to investigate plausible PA levels throughout pregnancy and examine how this natural variability associates with microbiota composition. Second, while age, pre-pregnancy BMI, and diet are critical biological factors that likely act as confounders, our primary models were adjusted only for race and marital status, as these were the only factors significantly associated with both the exposure and the outcome in this dataset. To address this, we conducted sensitivity analyses with “fully adjusted” models (**Supplementary Table 2**). Although high missingness in these covariates reduced the sample size by roughly two-thirds and limited statistical significance, the variance explained in these models remained notable. Third, the criterion of at least one valid day (≥10 hours of wear time) of PA may not be sufficient to capture daily variations in activity. Other studies, in non-pregnant populations, suggest that three to four days monitoring are needed to reliably estimate habitual physical activity (Hart et al., 2011; Chan et al., 2022). In a Brazilian population-based study of 2,082 pregnant women who wore an accelerometer for seven complete days, reliability to evaluate overall PA was reached with at least three monitoring days, whereas seven days were needed to estimate reliable measures of moderate-to-vigorous PA (Da Silva et al., 2019). We opted for a more exploratory approach for this preliminary investigation to maximize our analytic sample. It is important to note that the actual engagement was high; and because so few participants provided only a single day of data, this inclusive threshold is unlikely to have significantly impacted our findings. As data collection for this cohort is ongoing, future examinations with the full sample will utilize data-driven thresholds for inclusion that will be more robust than the predefined thresholds currently used in the literature. Fourth, microbiota composition was not measured longitudinally during pregnancy, and microbial function (such as with metagenomic sequencing) was not assessed. Finally, while the small sample size of the current study has its limits, it provides preliminary results that form a basis for hypotheses that can be tested in future studies as the larger population dataset becomes available.

## Conclusion

This study provides preliminary evidence that sedentary behavior during pregnancy is associated with differences in gut microbiota composition, including increased abundance of taxa previously linked to inflammation and metabolic dysfunction. These findings underscore the importance of reducing sedentary time during pregnancy and open new avenues for investigating how activity during gestation shapes the prenatal microbiome. Future studies should investigate whether specific microbial shifts associated with sedentary behavior, particularly *Acidaminococcus intestini* and *Oscillospirales* sp., are predictive of adverse pregnancy outcomes such as gestational diabetes or preeclampsia. Interventional studies focused on reducing sedentary time during pregnancy may provide causal or mechanistic insight. Additionally, integrating microbial function (e.g., SCFA production and metabolomics) will be critical for understanding the biological relevance of compositional shifts. Finally, research should explore whether microbial responses to PA are modified by diet, stress, or other environmental exposures during pregnancy. As research in this area grows, the maternal gut microbiome may emerge as a key mediator linking prenatal behavior to short- and long-term health outcomes for both mother and child.

## Data Availability

All raw sequences are deposited in the Sequence Read Archive (SRA) under Bioproject PRJNA1442233. All additional datasets and materials are available from the corresponding author upon reasonable request.
