# Supplementary material for: Association between objectively measured physical activity and maternal stool microbiota during pregnancy: results from a preliminary investigation
Source: Front Cell Infect Microbiol. 2026 Apr 23;16:1747305. doi: 10.3389/fcimb.2026.1747305 (PMC13149394; doi:10.3389/fcimb.2026.1747305)
Supplement: Supplementary file 1 [file SupplementaryFile1.pdf]

Supplemental Figures

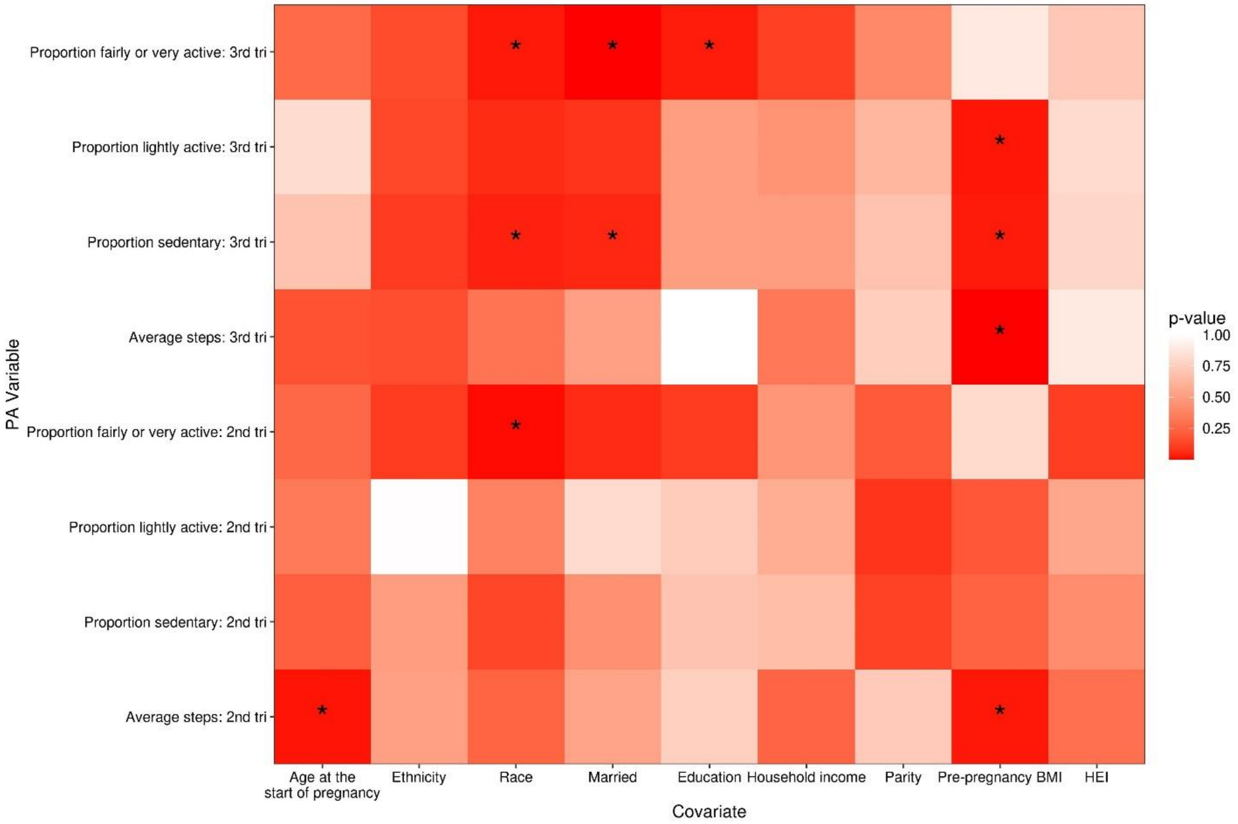

Supplemental Figure 1: Association between covariates and PA variables. P-values are calculated by the Kruskal-Wallis test for categorical covariates and Pearson correlations for continuous covariates. $P < 0.05$ are denoted by an asterisk. Abbreviations: tri, trimester; PA, physical activity; BMI, body mass index; HEI, healthy eating index.

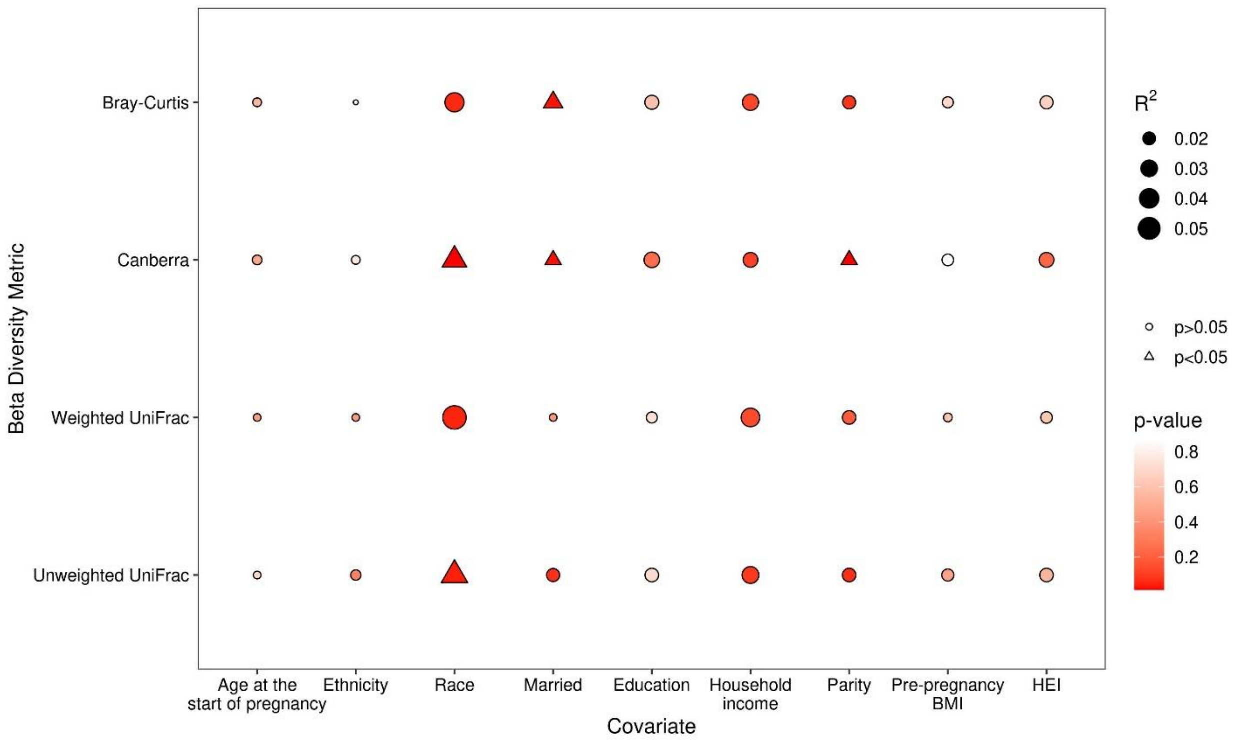

Supplemental Figure 2: Association between covariates and third trimester maternal stool composition. P-values and R^2 values are calculated using PERMANOVA. P < 0.05 are denoted by triangles. Abbreviations: BMI, body mass index; HEI, Health Eating Index; PERMANOVA, permutational multivariate analysis of variance.

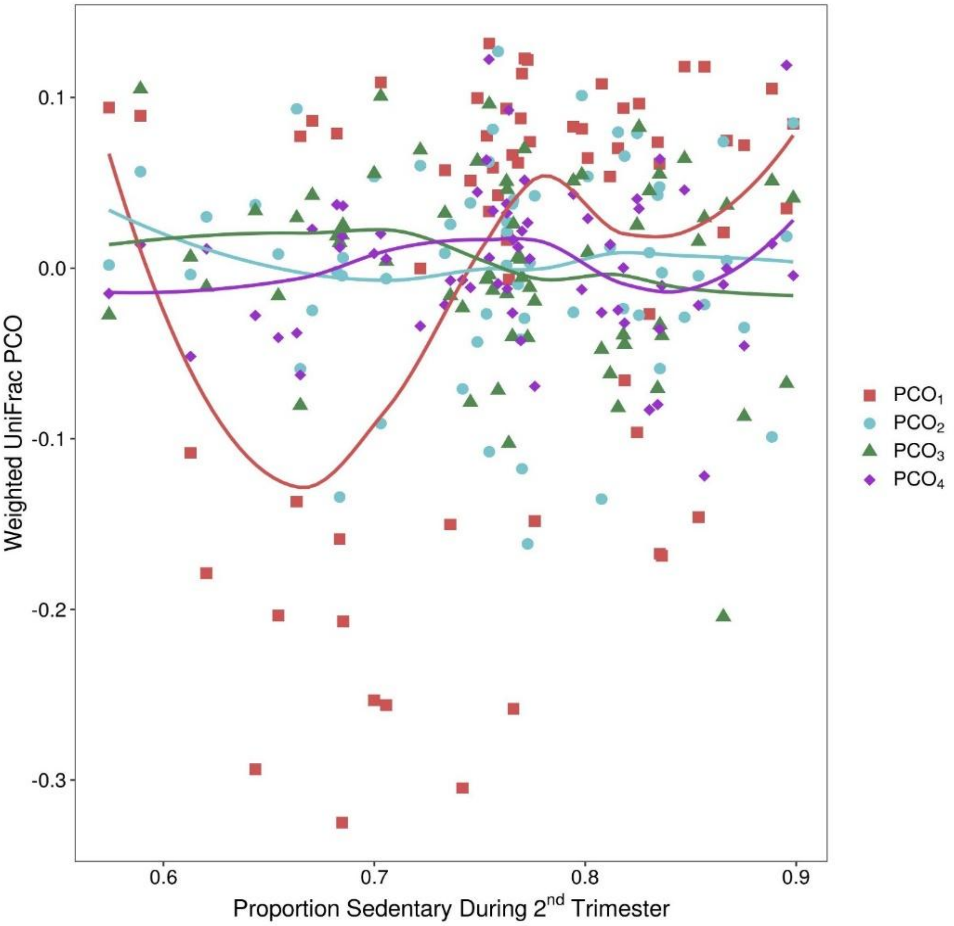

Supplemental Figure 3: Association between proportion sedentary during the second trimester and third trimester stool weighted UniFrac PCOs. Abbreviations: PCO, principal coordinates.

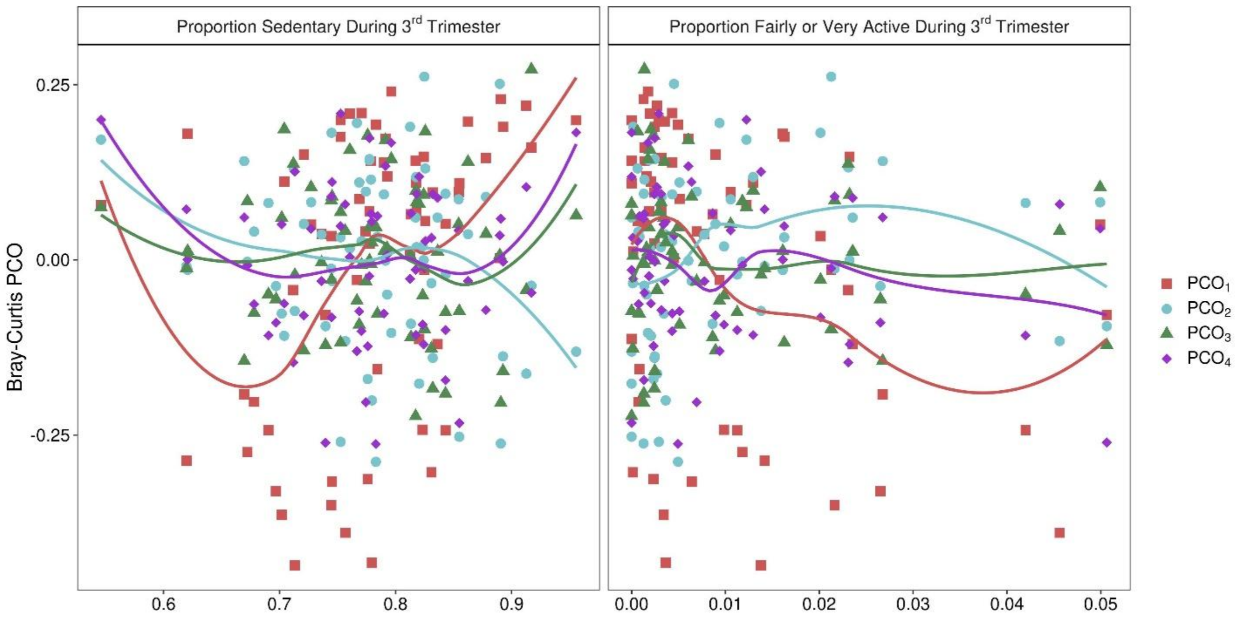

Supplemental Figure 4: Association between proportion sedentary and proportion fairly or very active during the third trimester and third trimester stool Bray-Curtis PCOs. Abbreviations: PCO, principal coordinates.

Supplemental Tables

Supplemental Table 1. Association between PA during the second and third trimester and stool beta diversity during the third trimester using non-phylogenetic metrics.

Beta diversity metric	PA trimester[a]	PA variable	Unadjusted		Adjusted[b]	
			p-value	R^2	p-value	R^2
Canberra	Second	Average steps	0.73	0.015	0.84	0.014
		Proportion sedentary	0.068	0.018	0.15	0.017
		Proportion lightly active	0.14	0.017	0.25	0.016
		Proportion fairly or very active	0.52	0.016	0.70	0.015
	Third	Average steps	0.60	0.016	0.70	0.015
		Proportion sedentary	**0.009**	0.021	0.050	0.019
		Proportion lightly active	**0.030**	0.02	0.097	0.018
		Proportion fairly or very active	**0.027**	0.02	0.10	0.018
Bray-Curtis	Second	Average steps	0.42	0.016	0.51	0.015
		Proportion sedentary	**0.015**	0.028	0.051	0.023
		Proportion lightly active	0.056	0.023	0.13	0.02
		Proportion fairly or very active	0.32	0.017	0.63	0.014
	Third	Average steps	0.34	0.017	0.42	0.016
		Proportion sedentary	**0.013**	0.031	**0.034**	0.025
		Proportion lightly active	**0.030**	0.027	0.084	0.022

Proportion fairly or very active	**0.006**	0.029	**0.047**	0.025

PA, physical activity.

[a]N=64 for all second trimester models; N=63 for all third trimester models.

[b]Adjusted for race and marital status.

Supplemental Table 2. Sensitivity analysis for the association between PA during the second and third trimester and stool beta diversity during the third trimester.

Beta diversity metric	PA trimester[a]	PA variable	Fully Adjusted[b]	
			p-value	R^2
Unweighted UniFrac	Second	Average steps	0.561	0.042
		Proportion sedentary	0.784	0.037
		Proportion lightly active	0.732	0.038
		Proportion fairly or very active	0.22	0.052
	Third	Average steps	0.961	0.029
		Proportion sedentary	0.985	0.027
		Proportion lightly active	0.992	0.027
		Proportion fairly or very active	0.228	0.049
Weighted UniFrac	Second	Average steps	0.711	0.032
		Proportion sedentary	0.81	0.027
		Proportion lightly active	0.788	0.028
		Proportion fairly or very active	0.78	0.028
	Third	Average steps	0.462	0.04
		Proportion sedentary	0.735	0.028
		Proportion lightly active	0.849	0.024

		Proportion fairly or very active	0.222	0.058
Canberra	Second	Average steps	0.688	0.043
		Proportion sedentary	0.774	0.042
		Proportion lightly active	0.723	0.042
		Proportion fairly or very active	0.188	0.048
	Third	Average steps	0.935	0.038
		Proportion sedentary	0.996	0.036
		Proportion lightly active	0.996	0.036
		Proportion fairly or very active	0.067	0.049
Bray-Curtis	Second	Average steps	0.75	0.039
		Proportion sedentary	0.625	0.042
		Proportion lightly active	0.531	0.045
		Proportion fairly or very active	0.566	0.043
	Third	Average steps	0.79	0.036
		Proportion sedentary	0.859	0.034
		Proportion lightly active	0.878	0.033
		Proportion fairly or very active	0.064	0.062

PA, physical activity.

[a]N=23 for all second trimester models; N=24 for all third trimester models.

[b]Adjusted for age at the start of pregnancy, ethnicity, race, marital status, education, household income, parity, pre-pregnancy BMI, and HEI.
