# Supplementary material for: Association between objectively measured physical activity and maternal stool microbiota during pregnancy: results from a preliminary investigation
Source: Front Cell Infect Microbiol. 2026 Apr 23;16:1747305. doi: 10.3389/fcimb.2026.1747305 (PMC13149394; doi:10.3389/fcimb.2026.1747305)
Supplement: Supplementary file 2 [file Table1.docx]

**Supplemental Figures**


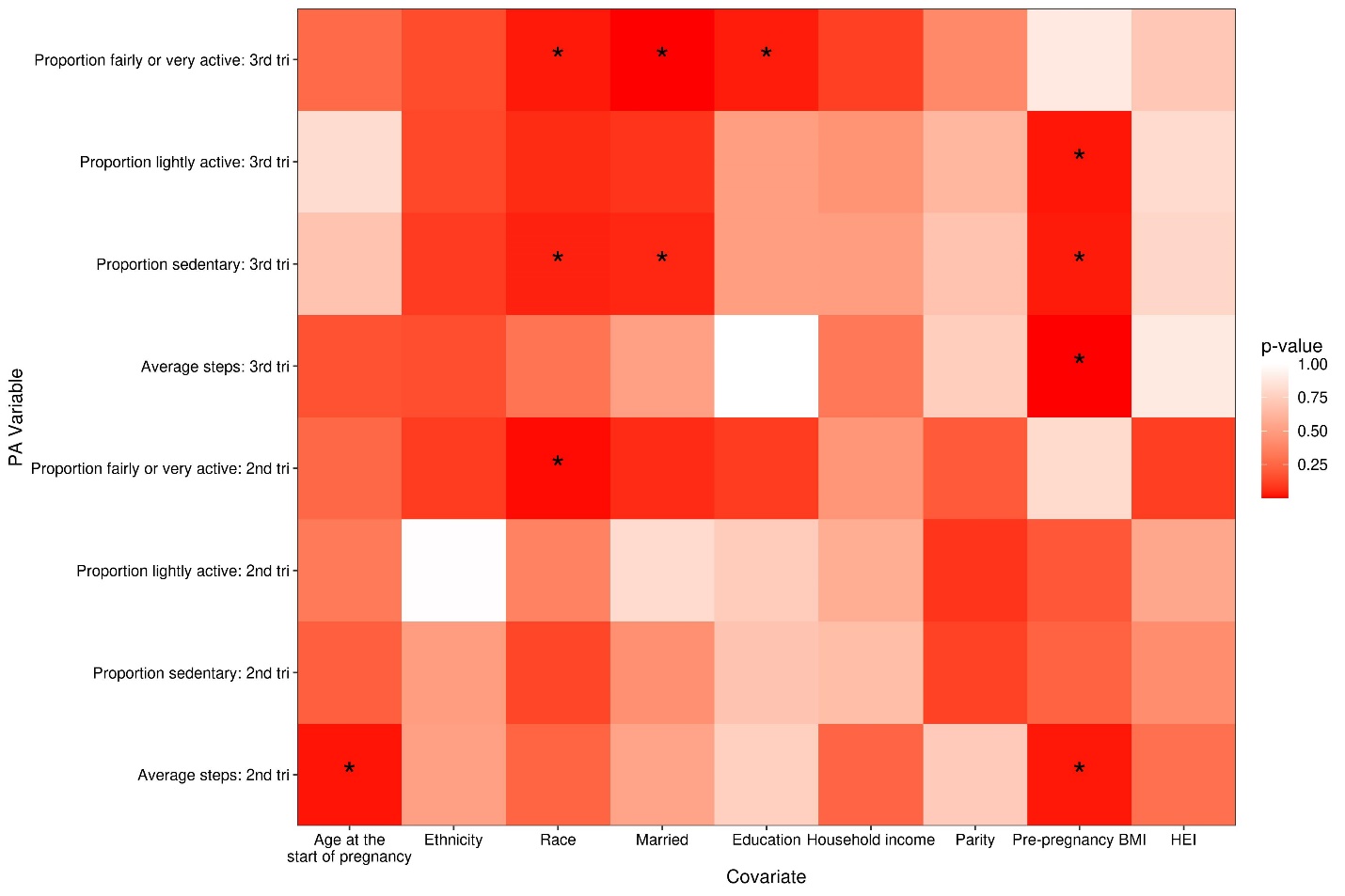


Supplemental Figure 1: Association between covariates and PA variables. P-values are calculated by the Kruskal-Wallis test for categorical covariates and Pearson correlations for continuous covariates. P < 0.05 are denoted by an asterisk. Abbreviations: tri, trimester; PA, physical activity; BMI, body mass index; HEI, healthy eating index.


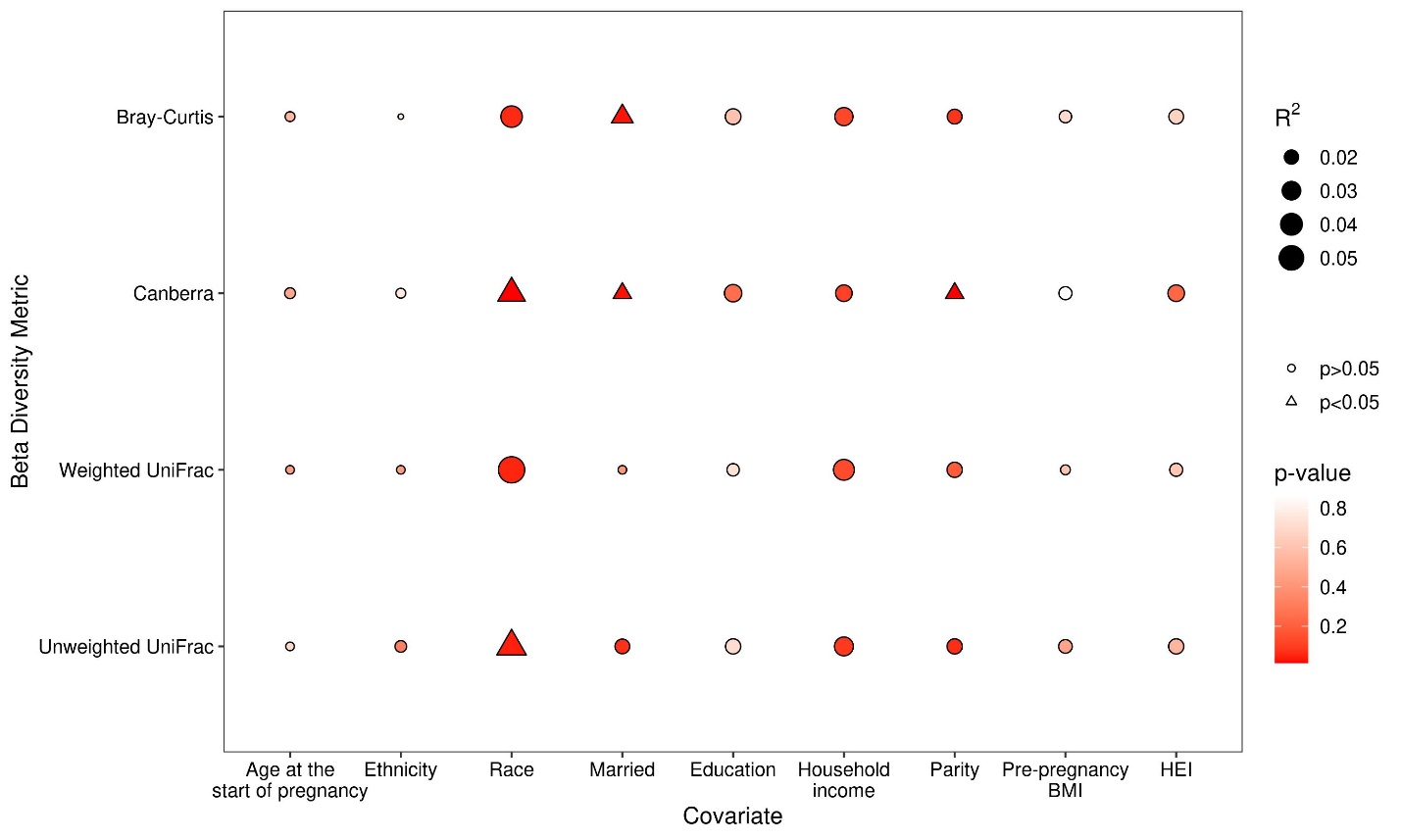


Supplemental Figure 2: Association between covariates and third trimester maternal stool composition. P-values and R^2^ values are calculated using PERMANOVA. P < 0.05 are denoted by triangles. Abbreviations: BMI, body mass index; HEI, Health Eating Index; PERMANOVA, permutational multivariate analysis of variance.


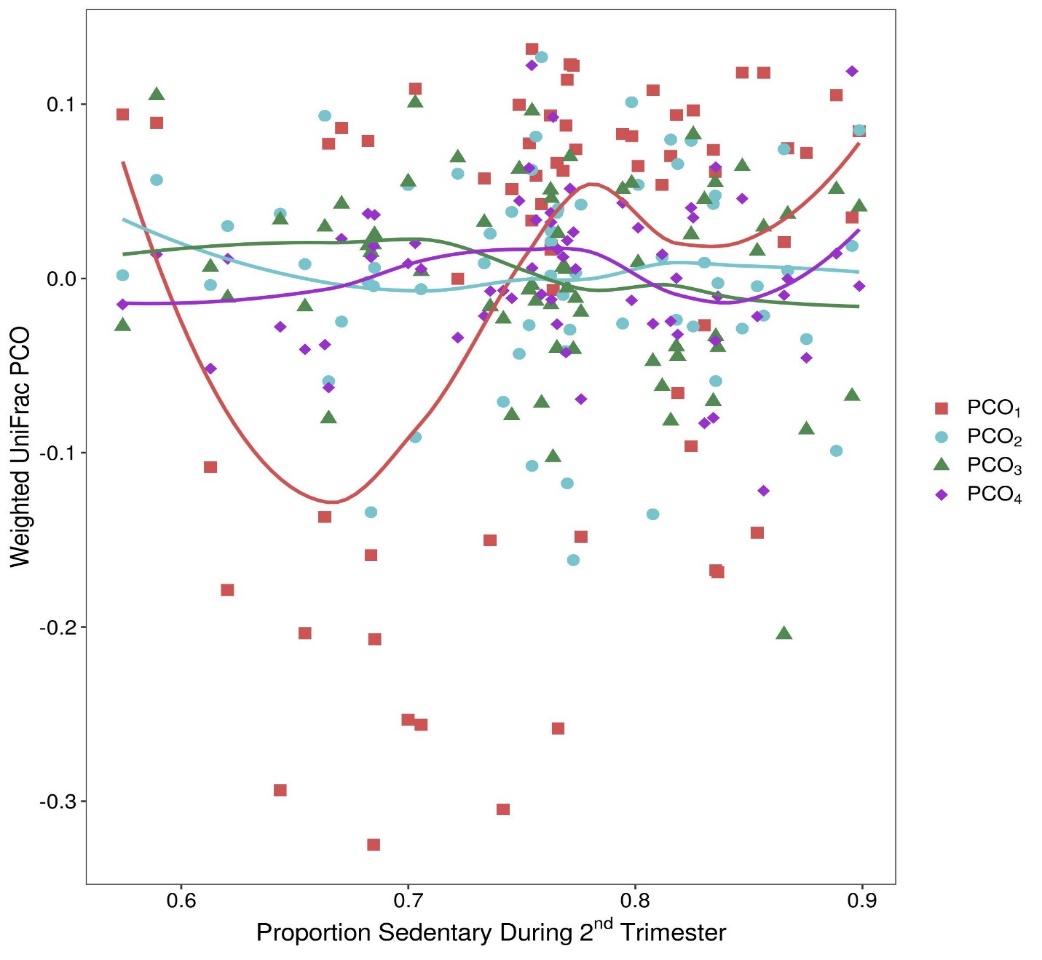


Supplemental Figure 3: Association between proportion sedentary during the second trimester and third trimester stool weighted UniFrac PCOs. Abbreviations: PCO, principal coordinates.


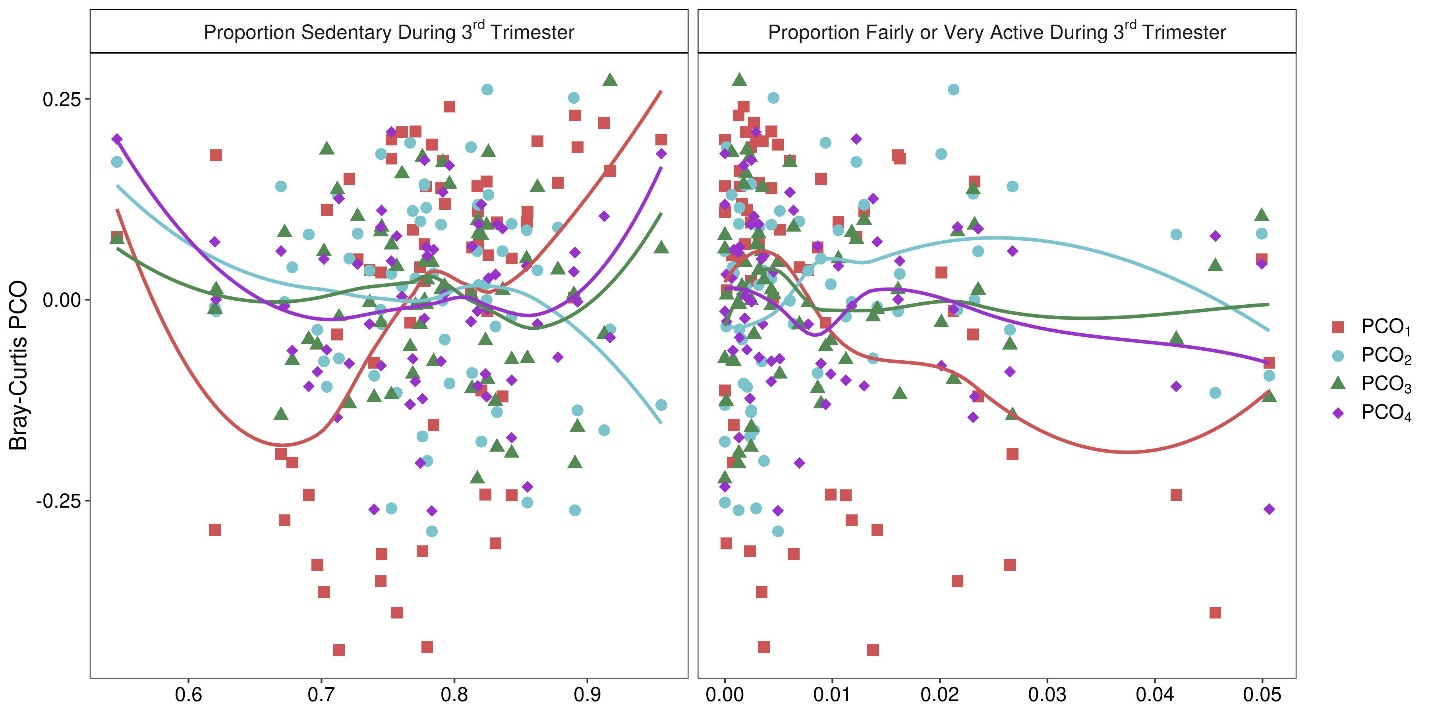


Supplemental Figure 4: Association between proportion sedentary and proportion fairly or very active during the third trimester and third trimester stool Bray-Curtis PCOs. Abbreviations: PCO, principal coordinates.

**Supplemental Tables**

**Supplemental Table 1. Association between PA during the second and third trimester and stool beta diversity during the third trimester using non-phylogenetic metrics.**

| **Beta diversity metric** | **PA trimester^a^** | **PA variable** | **Unadjusted** | | **Adjusted^b^** | |
| --- | --- | --- | --- | --- | --- | --- |
|  |  |  | **p-value** | **R^2^** | **p-value** | **R^2^** |
| Canberra | Second | Average steps | 0.73 | 0.015 | 0.84 | 0.014 |
|  |  | Proportion sedentary | 0.068 | 0.018 | 0.15 | 0.017 |
|  |  | Proportion lightly active | 0.14 | 0.017 | 0.25 | 0.016 |
|  |  | Proportion fairly or very active | 0.52 | 0.016 | 0.70 | 0.015 |
|  | Third | Average steps | 0.60 | 0.016 | 0.70 | 0.015 |
|  |  | Proportion sedentary | **0.009** | 0.021 | 0.050 | 0.019 |
|  |  | Proportion lightly active | **0.030** | 0.02 | 0.097 | 0.018 |
|  |  | Proportion fairly or very active | **0.027** | 0.02 | 0.10 | 0.018 |
| Bray-Curtis | Second | Average steps | 0.42 | 0.016 | 0.51 | 0.015 |
|  |  | Proportion sedentary | **0.015** | 0.028 | 0.051 | 0.023 |
|  |  | Proportion lightly active | 0.056 | 0.023 | 0.13 | 0.02 |
|  |  | Proportion fairly or very active | 0.32 | 0.017 | 0.63 | 0.014 |
|  | Third | Average steps | 0.34 | 0.017 | 0.42 | 0.016 |
|  |  | Proportion sedentary | **0.013** | 0.031 | **0.034** | 0.025 |
|  |  | Proportion lightly active | **0.030** | 0.027 | 0.084 | 0.022 |
|  |  | Proportion fairly or very active | **0.006** | 0.029 | **0.047** | 0.025 |

PA, physical activity.

^a^N=64 for all second trimester models; N=63 for all third trimester models.

^b^Adjusted for race and marital status.

**Supplemental Table 2. Sensitivity analysis for the association between PA during the second and third trimester and stool beta diversity during the third trimester.**

| **Beta diversity metric** | **PA trimester^a^** | **PA variable** | **Fully Adjusted^b^** | |
| --- | --- | --- | --- | --- |
|  |  |  | **p-value** | **R^2^** |
| Unweighted UniFrac | Second | Average steps | 0.561 | 0.042 |
|  |  | Proportion sedentary | 0.784 | 0.037 |
|  |  | Proportion lightly active | 0.732 | 0.038 |
|  |  | Proportion fairly or very active | 0.22 | 0.052 |
|  | Third | Average steps | 0.961 | 0.029 |
|  |  | Proportion sedentary | 0.985 | 0.027 |
|  |  | Proportion lightly active | 0.992 | 0.027 |
|  |  | Proportion fairly or very active | 0.228 | 0.049 |
| Weighted UniFrac | Second | Average steps | 0.711 | 0.032 |
|  |  | Proportion sedentary | 0.81 | 0.027 |
|  |  | Proportion lightly active | 0.788 | 0.028 |
|  |  | Proportion fairly or very active | 0.78 | 0.028 |
|  | Third | Average steps | 0.462 | 0.04 |
|  |  | Proportion sedentary | 0.735 | 0.028 |
|  |  | Proportion lightly active | 0.849 | 0.024 |
|  |  | Proportion fairly or very active | 0.222 | 0.058 |
| Canberra | Second | Average steps | 0.688 | 0.043 |
|  |  | Proportion sedentary | 0.774 | 0.042 |
|  |  | Proportion lightly active | 0.723 | 0.042 |
|  |  | Proportion fairly or very active | 0.188 | 0.048 |
|  | Third | Average steps | 0.935 | 0.038 |
|  |  | Proportion sedentary | 0.996 | 0.036 |
|  |  | Proportion lightly active | 0.996 | 0.036 |
|  |  | Proportion fairly or very active | 0.067 | 0.049 |
| Bray-Curtis | Second | Average steps | 0.75 | 0.039 |
|  |  | Proportion sedentary | 0.625 | 0.042 |
|  |  | Proportion lightly active | 0.531 | 0.045 |
|  |  | Proportion fairly or very active | 0.566 | 0.043 |
|  | Third | Average steps | 0.79 | 0.036 |
|  |  | Proportion sedentary | 0.859 | 0.034 |
|  |  | Proportion lightly active | 0.878 | 0.033 |
|  |  | Proportion fairly or very active | 0.064 | 0.062 |

PA, physical activity.

^a^N=23 for all second trimester models; N=24 for all third trimester models.

^b^Adjusted for age at the start of pregnancy, ethnicity, race, marital status, education, household income, parity, pre-pregnancy BMI, and HEI.
